# NURSING HOME BARRIERS AND ENABLERS TO USING THE GOALS OF CARE VIDEO INTERVENTION

**DOI:** 10.1093/geroni/igae098.3873

**Published:** 2024-12-31

**Authors:** Latarsha Chisholm, Susan Hickman, Kathleen Unroe

**Affiliations:** University of Central Florida, Orlando, Florida, United States; Regenstrief Institute, Inc., Indianapolis, Indiana, United States; Indiana University, Indianapolis, Indiana, United States

## Abstract

Advance care planning (ACP) is a process that prepares individuals for communicating and making decisions about personal goals regarding medical care. Despite the benefits, ACP is limited across nursing homes (NHs) due to implementation challenges. It is essential to understand how to implement ACP, like Goals of Care (GOC) evidence-based intervention. The GOC video is intended to supplement ACP discussions to educate patients living with dementia and families. This study was performed to identify enablers and barriers to using the GOC video. We interviewed 12 staff from six Florida NHs. A pre-implementation guide was developed using constructs from the Consolidated Framework for Implementation Research 2.0. Enablers included: mission alignment (6 out of 6 NHs); physical space, use of information technology, and learning-centeredness (5 out of 6 NHs); and work infrastructure, communication, readiness, recipient-centeredness, and implementation facilitators (4 out of 6 NHs). Barriers included: design of the GOC video (6 out of 6 NHs; access to health education (5 out of 6 NHs); and patient readiness and culture as barriers (4 out of 6 NHs). NH staff reported various barriers and enablers to use the GOC video; however, some barriers and enablers were similar across NHs. Developing a standardized protocol that allows flexibility maybe helpful to nursing homes implementing the GOC video intervention.

